# A Network Pharmacology Approach to Uncover the Molecular Mechanisms of Herbal Formula Ban-Xia-Xie-Xin-Tang

**DOI:** 10.1155/2018/4050714

**Published:** 2018-10-16

**Authors:** Ming Yang, Jialei Chen, Liwen Xu, Xiufeng Shi, Xin Zhou, Rui An, Xinhong Wang

**Affiliations:** ^1^Department of Pharmacy, Longhua Hospital Affiliated to Shanghai University of TCM, Shanghai, China; ^2^Department of Chemistry, College of Pharmacy, Shanghai University of Traditional Chinese Medicine, Shanghai, China

## Abstract

Ban-Xia-Xie-Xin-Tang (BXXXT) is a classical formula from Shang-Han-Lun which is one of the earliest books of TCM clinical practice. In this work, we investigated the therapeutic mechanisms of BXXXT for the treatment of multiple diseases using a network pharmacology approach. Here three BXXXT representative diseases (colitis, diabetes mellitus, and gastric cancer) were discussed, and we focus on in silico methods that integrate drug-likeness screening, target prioritizing, and multilayer network extending. A total of 140 core targets and 72 representative compounds were finally identified to elucidate the pharmacology of BXXXT formula. After constructing multilayer networks, a good overlap between BXXXT nodes and disease nodes was observed at each level, and the network-based proximity analysis shows that the relevance between the formula targets and disease genes was significant according to the shortest path distance (SPD) and a random walk with restart (RWR) based scores for each disease. We found that there were 22 key pathways significantly associated with BXXXT, and the therapeutic effects of BXXXT were likely addressed by regulating a combination of targets in a modular pattern. Furthermore, the synergistic effects among BXXXT herbs were highlighted by elucidating the molecular mechanisms of individual herbs, and the traditional theory of “Jun-Chen-Zuo-Shi” of TCM formula was effectively interpreted from a network perspective. The proposed approach provides an effective strategy to uncover the mechanisms of action and combinatorial rules of BXXXT formula in a holistic manner.

## 1. Introduction

Traditional Chinese medicine (TCM) has been widely used in China for thousands of years. Understanding the mechanisms of TCM helps to improve its clinical application. Therefore, there has been considerable interest in the development of experimental technologies to discover relationships between biological processes and TCM treatment, such as high-throughput biological profiling [1]. Recent advances in this field result in large accumulated data, and more powerful tools and approaches should be needed to achieve a comprehensive analysis by integrating systematic information to contextualize the holistic characteristic of TCM rather than focus on highly selective agents targeting specific proteins. Recently, a network pharmacology approach has been proposed, and the overall analysis strategy has been shifting away from “one drug–one target–one disease” to the idea of binding multiple targets [2–6]. Li [7] presented a novel concept of “network target” that regards the biological network as a target through which the best drug intervention can be designed and developed. His group introduced this network-based methodology to investigate the basic biological knowledge underling TCM [8, 9]. Network pharmacology provides a deeper insight or scientific evidence for TCM knowledge and helps us elucidate action mechanisms at a biological molecular level. Recent advances in TCM network pharmacology research have demonstrated the systematic mechanisms of TCM treatment for complex diseases [10–14]. Several classical herbal formulas [15–18] were identified as scientifically proven in a systematic manner, as well as their combinatorial rules. For example, Liang et al. [18] applied a network analysis to point out the mechanism of Liu-Wei-Di-Huang pill for the treatments of different diseases. The key biological processes of Qing-Luo-Yin against rheumatoid arthritis were revealed by the integrative TCM network pharmacology platform [11]. Li et al. [19] performed a system-level investigation into the mechanisms of compound Danshen formula for the treatment of cardiovascular disease by integrating oral bioavailability screening and molecular docking techniques.

Ban-Xia-Xie-Xin-Tang (BXXXT) [20] is a classical formula from Shang-Han-Lun which is one of the earliest books of TCM clinical practice. BXXXT consists of seven herbs:* Pinellia ternata* (Thunb.) Makino (Ban-Xia in Chinese, BX),* Zingiber officinale* Roscoe (Gan-Jiang in Chinese, GJ),* Coptis chinensis* Franch. (Huang-Lian in Chinese, HL),* Scutellaria baicalensis* Georgi (Huang-Qin in Chinese, HQ), Panax ginseng C.A.Mey. (Ren-Shen in Chinese, RS),* Ziziphus jujuba* Mill. (Da-Zao in Chinese, DZ), and* Glycyrrhiza uralensis* Fisch. (Gan-Cao in Chinese, RGC). BXXXT has been widely used in TCM clinical practice. Previous studies [20, 21] have illustrated that BXXXT possesses anti-inflammatory activities, and it can be used for the treatments of various digestive inflammations such as colitis, esophagitis, and gastritis [20, 22]. On the other hand, multiple and diverse indications that extend the applications of original context were reported for the use of BXXXT. These extensive indications include functional dyspepsia [21], diabetes mellitus (DM)[23], gastric cancer (GC) [24], and cardiovascular disease [25, 26]. Although some targets and pathways were examined in the past few decades [27–29], the biological mechanism of BXXXT in a holistic manner remains unknown because of its complex nature. To better understand its molecular basis of “one formula, different diseases”, in this work, a network pharmacology approach was proposed to uncover the mechanisms of BXXXT for the treatments of three representative diseases including colitis, DM, and GC. Herein we focus on in silico methods, and the main contributions of our work include the following. (1) Chemical characteristics of BXXXT compounds were analyzed, and the active compounds of the formula were recognized by using a drug-likeness measure. Then an overrepresentation analysis was performed to determine the core targets of the formula, and the core ingredients were identified after a target score function was defined. (2) Multilayer networks were constructed based on the core targets and disease (colitis, DM, and GC) genes, respectively. Comparisons of network statistics were performed to better understand the biological profiles. Furthermore, two network-based proximity measures were used to evaluate relationships between ingredient targets and disease genes. (3) Biological functional annotation analysis was performed on ingredient targets and disease genes, respectively. Then common biological entities and processes between diseases and the formula could be achieved to facilitate the analysis of molecular mechanisms of BXXXT for the treatments of multiple diseases. Our results show that there were good overlaps between formula-related network and disease-related network at each layer level, and BXXXT treats multiple diseases probably by regulating some potential shared biological processes. This work provides a new insight into the biological mechanisms of BXXXT and promotes the drug development based on this formula.

## 2. Materials and Methods

### 2.1. Data Preparation

#### 2.1.1. Ingredients of BXXXT and Their Targets

There are seven herbs in BXXXT, and the compounds contained in these herbs can be considered as ingredients of BXXXT. Ingredients of BXXXT were collected from four data sources: (1) Chinese academy of sciences Chemistry Database [19], (2) HIT database [30, 31], (3) TCMID database [30, 32], and (4) traditional Chinese medicine information database [33]. All compounds were processed as follows: (1) they were represented by SMILES format and were prepared by Prepare Ligand Module in Discovery Studio 2.5 (DS2.5) and (2) duplicates were removed. A total of 560 unique compounds were obtained. In addition to the above data sources, STITCH database [34] was used to retrieve compound targets. This leads to a data set of 4680 interactions between 194 compounds and 2213 targets. All compounds and their targets are available in Supporting Information Table S1.

#### 2.1.2. Disease Associated Genes

In this work, colitis, DM, and GC were considered as BXXXT diseases, and their associated genes were collected from the Online Mendelian Inheritance in Man (OMIM) database [35] and therapeutic target database (TTD) [36], respectively. As a result, we obtained 28, 119, and 30 genes associated with colitis, DM, and GC, respectively. All disease associated genes are available in Supporting Information Table S2.

#### 2.1.3. FDA-Approved Drugs and Their Targets

The FDA-approved drugs for the treatment of colitis, DM, and GC and their targets were extracted from the DrugBank database [37]. The results are as follows: (1) 9 drugs and 21 targets for colitis, (2) 40 drugs and 43 targets for DM, and (3) 8 drugs and 19 targets for GC. The detailed information of these drugs and targets are available in Supporting Information Table S3.

#### 2.1.4. Protein-Protein Interaction Data

In this work, protein-protein interaction (PPI) data were imported from Human Integrated Protein-Protein Interaction rEference (HIPPIE) database [38, 39]. HIPPIE has integrated interactions from some studies [38, 39] and multiple public PPI databases such as HPRD [40], BioGRID [41], IntAct [42], MINT [43], DIP [44], and BIND [45]. Furthermore, confidence scores of interactions are presented in HIPPIE to signify the reliability of their experimental evidences. In total, HIPPIE (version 1.8) consists of 239685 interactions. To obtain high-confidence interactions, we have removed the bottom scored interactions (score = 0). This leads to a PPI data concerning 16503 genes and 230434 interactions.

### 2.2. Chemical Characteristics of BXXXT

Oral administration is the main route of administration for TCM. However, it is limited by the drug's ADME (absorption, distribution, metabolism, and elimination) characteristics [12, 46, 47]. The poor ADME properties are largely accountable for drug failure in exerting pharmacodynamic effect on target site in vivo [12]. Drug-likeness is usually used to signify whether the compound has acceptable ADME properties. The assessment of drug-likeness of compounds helps to identify the bioactive ingredients in TCM formulas [12, 48]. In this work, the quantitative estimate of drug-likeness (QED) presented by Bickerton [49] was used to prescreen pharmaceutically active compounds in BXXXT. This measure of drug-likeness is calculated by integrating eight physicochemical properties of molecules [49]: (1) molecular mass, (2) number of hydrogen bond donors (HBDs), (3) octanol–water partition coefficient (ALOGP), (4) number of hydrogen bond acceptors (HBAs), (5) number of rotatable bonds (ROTBs), (6) number of aromatic rings (AROMs), (7) molecular polar surface area (PSA), and (8) number of structural alerts (ALERTS). Our previous work suggested that QED values can be used for the assessment of some ADME characteristics straightforwardly [46].

### 2.3. Target Profiling of BXXXT Ingredients

The core targets of BXXXT can be determined by the analysis of target profiling of BXXX ingredients. The current work is strongly motivated by the previously published approach [18] for the analysis of core targets. The target that interacts with many compounds can be regarded as a core target in the pharmacological effects of the formula. Then, the probability of a target being core target was evaluated as follows using a binomial statistical model:

(1) $$\begin{array}{c} P\left(\;X\geq k\;\right)=\overset{n}{\underset{m=k}{\sum }}C_{n}^{m}{\left(\;\frac{g}{n}\;\right)}^{m}{\left(\;1-\frac{g}{n}\;\right)}^{n-m} \end{array}$$

where *k* is the number of compounds interacting with the investigated target, *n* is the total number of compounds, and *g* denotes the average number of compounds per target. Then *P*(*X* ≥ *k*), after being adjusted by the false discovery rate method, measures the probability of a target interacting with more than *k* compounds in the profile of *n* compounds by random chance. These statistics help to estimate how likely that the result is stochastic. The target with a low *P* value (*P* value < 0.05), which means that the observed number of interacting compounds is significantly larger than the expected one, can be considered as a core target for the formula.

Then the score for a specific target (GS) was assessed as follows:

(2) $$\begin{array}{c} GS=\left\{\begin{array}{cc} \frac{-\log\left(P\left(X\geq k\right)\right)}{Rank\left(P\left(X\geq k\right)\right)}, & if\;\;P\left(X\geq k\right)\leq 0.05\\ 0, & otherwise \end{array}\right. \end{array}$$

where *GS* of the core target was calculated by use of a numerator that equaled the negative logarithm of *P*(*X* ≥ *k*) and a denominator that equaled the rank of *P*(*X* ≥ *k*); otherwise it equaled zero. In order to identify the core ingredients of BXXXT, the score of a compound (CS) was calculated by averaging its corresponding target scores:

(3) $$\begin{array}{c} CS_{i}=\frac{1}{N_{i}}\overset{N_{i}}{\underset{j=1}{\sum }}GS_{j} \end{array}$$

where *N*_*i*_ represents the number of interacting targets for compound *i* and *GS*_*j*_ is the score of target *j* in the target profile of compound *i*. *GS*_*j*_ can be calculated using (2).

### 2.4. Network Construction and Analysis

#### 2.4.1. Network Construction

In this work, we consider the core targets of the formula as formula targets. In order to elucidate the biological profiles of BXXXT at different levels, multilayer networks were built based on formula targets and disease (colitis, DM, and GC) genes, respectively. These multilayer networks mainly include three levels as follows.

1. Core net (CN) level: here we consider the formula targets or disease genes as query genes. The query genes were mapped to PPI network. Then the CN of the formula and disease was generated from a subnet containing only the query genes and all the edges among them, respectively.
2. Shortest path extending net (SPEN) level: it is assumed that the intermediate genes which are along the shortest paths between all pairs of query genes in PPI network may have a high probability of being important roles for the coregulation of query genes [50]. The SPEN was generated by adding the intermediate genes, and the extension steps were performed as follows: first, the lengths of pairwise shortest paths between query genes were calculated. To reduce the high-false positives on the long paths, those paths whose lengths were no greater than 3 were picked out for investigation. Second, the query genes were updated by adding the genes along the satisfying paths. Finally, The SPEN of the formula and disease was generated by the same way that the CN was built.
3. Neighbor extending net (NEN) level: the query genes were updated by adding their neighbors in PPI network. Then the NEN of the formula and disease was generated by the same way that the CN was built.

**Figure 1:**
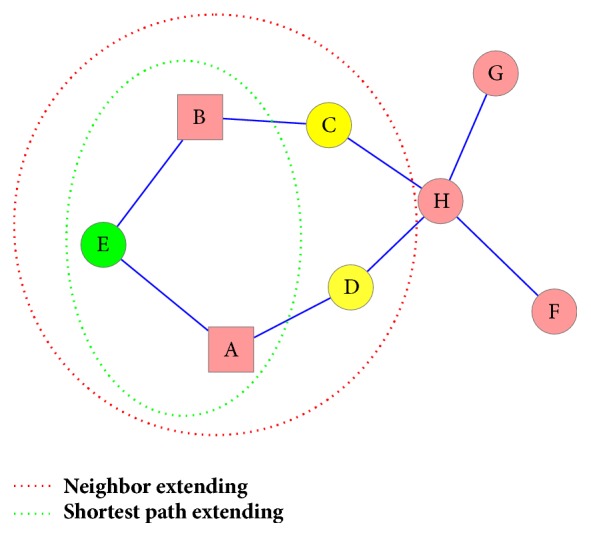
Illustrative example of network extending.

As shown in Figure 1, for example, there is a simple PPI network consisting of eight genes, named Genes A~H, respectively. Gene A and Gene B are query genes. Then the CN contains only Gene A and Gene B. The length of shortest path between A and B is 2 (≤3), and Gene E on this path is selected for extending the network. The SPEN contains Gene A, Gene B, and Gene E, and all the edges among them. Gene A's neighbors (E and D) and Gene B's neighbors (E and C), together with the query genes, are used for the NEN construction. After multilayer network construction, descriptive analysis of network characteristics was performed for the formula networks and disease networks, respectively. Besides, a similarity score function was defined as follows to measure the degree of node overlap between networks.

(4) $$\begin{array}{c} \mathrm{Sim}\left(\;Net_{A},Net_{B}\;\right)=\frac{Count\left(N_{A}\cap N_{B}\right)}{\min\left(Count\left(N_{A}\right),Count\left(N_{B}\right)\right)} \end{array}$$

where N_A_ and N_B_ represent the nodes of Network A and Network B, respectively. Count is a counting function to calculate the number of common nodes between networks, and min is a function to calculate the minimum number of nodes in networks.

#### 2.4.2. Functional Profile Analysis

For functional analysis, enrichment analysis was implemented to identify whether a gene ontology (GO) term or a pathway was significantly associated with the formula targets and disease genes, respectively. A hypergeometric test was used to estimate the association of annotation terms to the query genes, and the probability of getting at least *k* genes from the reference list by chance can be calculated as follows [51].

(5) $$\begin{array}{c} P=1-\overset{k-1}{\underset{i=0}{\sum }}\frac{\left(\begin{array}{c} M\\ i \end{array}\right)\left(\begin{array}{c} N-M\\ n-i \end{array}\right)}{\left(\begin{array}{c} N\\ n \end{array}\right)} \end{array}$$

where *N* is the total number of genes from the reference terms, *M* represents the number of genes annotated by a specific reference term (GO or pathway), *n* is the number of query genes, and *k* denotes the number of common genes between query genes and the reference set. *P* was adjusted by the false discovery rate method, and the low adjusted value (<0.01) indicate the significant association. In this work, GO enrichment analysis for molecular function (MF), cellular component (CC), and biological process (BP) was performed, and pathway enrichment analysis was based on KEGG pathway database[52].

#### 2.4.3. Network-Based Proximity Measures

Generally speaking, the network-based relevance inference methods make use of information from the network topological structure [53, 54]. It is important to consider the relevance between the formula targets and disease genes for the elucidation of the associations between the formula and disease. The basic assumption is that if the formula is effective for the treatment of the disease, the formula targets are likely to be close to disease genes in the PPI network [55]. In this study, two network-based proximity measure approaches were used to capture such relatedness: the shortest path distance (SPD)[56] and a random walk with restart (RWR)[57] based scoring method.

The shortest path between genes is a path with the minimal number of genes, and the distance between genes is the number of edges in a shortest path linking them. The average SPD between the formula targets and disease genes in the PPI network was calculated. The RWR method is a diffusion based approach [57]. In this approach, a set of random walkers were simulated. They started at disease genes and moved to their neighbors randomly at each step. Then the probability of the random walkers hitting the formula targets at step *t* could be calculated iteratively as follows.

(6) $$\begin{array}{c} x^{t+1}=\left(\;1-\gamma \;\right)Mx^{t}+\gamma x^{0} \end{array}$$

where *x*^0^, *x*^*t*^, and *x*^*t*+1^ represent the probability of finding the random walkers at the formula targets at initial state, step *t*, and step *t*+1, respectively. *M* is the transition matrix of the PPI network, and *γ* is the restart probability with which the random walkers can return to the disease genes. Here, *x*^0^ represents the initial probability of the random walkers hitting the formula targets, and *x*^0^ was denoted by a vector with equal probabilities assigned to disease genes in the PPI network. In this work, *γ* was set to 0.7, and the initial probabilities of disease genes were all set to 1 and others were all set to zero. A stable state will be reached if the difference between *x*^*t*^ and *x*^*t*+1^ falls below 10^−10^ after some steps. Then *x*^*∞*^ can be considered as a proximity score for measuring the closeness between formula targets and disease genes.

#### 2.4.4. Network Module Analysis

A network module can be viewed as a group of densely connected nodes in the interactome network. Identification of gene modules can provide better understanding of the underlying mechanisms. With the increasing application of network technique, several methods have been devised and implemented to detect communities of a complex network[58–66]. In this work, five different network module identification methods were explored and compared: (1) the fast greedy (FG) modularity based algorithm [59], (2) molecular complex detection (MCODE) [63], (3) neighbor-sharing score with hierarchical agglomerative clustering for module identification (NeMo) [64], (4) Mofinder[65], and (5) IPCA[66]. A scoring scheme was developed to measure the goodness of network partition. This module score was defined as *MS*:

(7) $$\begin{array}{c} MS=Q+BHI \end{array}$$

where *Q* is the modularity of the divided network and *Q* was calculated as (8) [59] in which *E* is the number of edges, *AM* represents the network adjacency matrix, *D* is the node degree of gene, and *δ* denotes an indicator showing whether the two genes belong to the same module. According to the definition above, *Q* measures the degree of density for the module. *BHI* is a biological homogeneity index that measures the functional homogeneity of genes within a module, and *BHI* was calculated using (9) [67] in which *K* is the number of modules, *n*_*k*_ is the total number of the genes in module *k* annotated in KEGG database, and *I* denotes an indicator showing whether the two genes belong to the same KEGG pathway. More details about this metric have been described elsewhere [67]. In our scoring scheme, both metrics fall in the range of 0 to 1, and a large *MS* value indicates a good network partition method. Then the best network module identification method was determined and implemented, and the functional modules were further analyzed to explore the molecular mechanisms.

(8) Q=12E∑ijAMij−DiDj2EδCi,Cj

(9) BHIM,B=1K∑k=1K∑i≠j∈MkIBi==Bjnknk−1

### 2.5. Tools

All compounds were prepared by Prepare Ligand Module in Discovery Studio 2.5, and PaDEL[68] 2.20 was used to calculate the drug-likeness descriptors. All network analysis was implemented using R 3.4.3, and Cytoscape[69] 3.6.1 was used to visualize the network.

## 3. Results and Discussion

### 3.1. Chemical Characteristics of BXXXT Compounds

**Figure 2:**
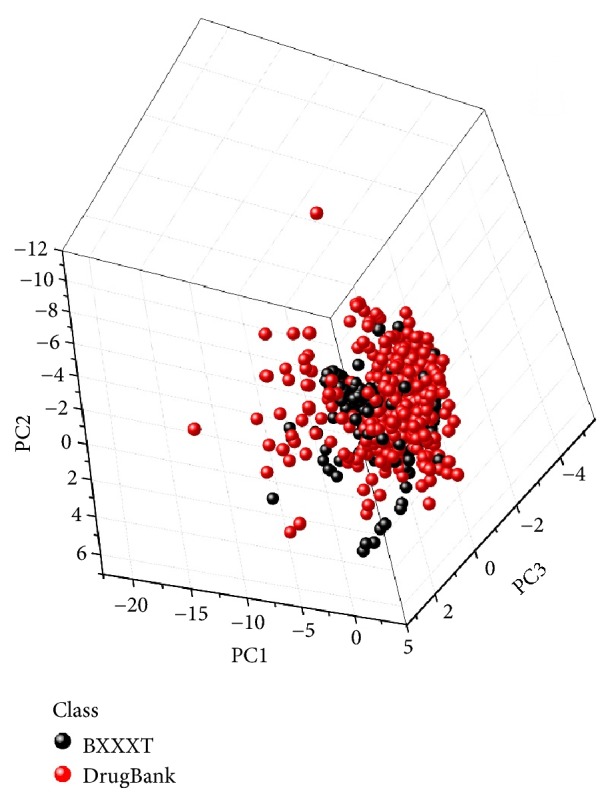
Score plot from PCA based on the combination of BXXXT compounds and DrugBank drugs.

**Figure 3:**
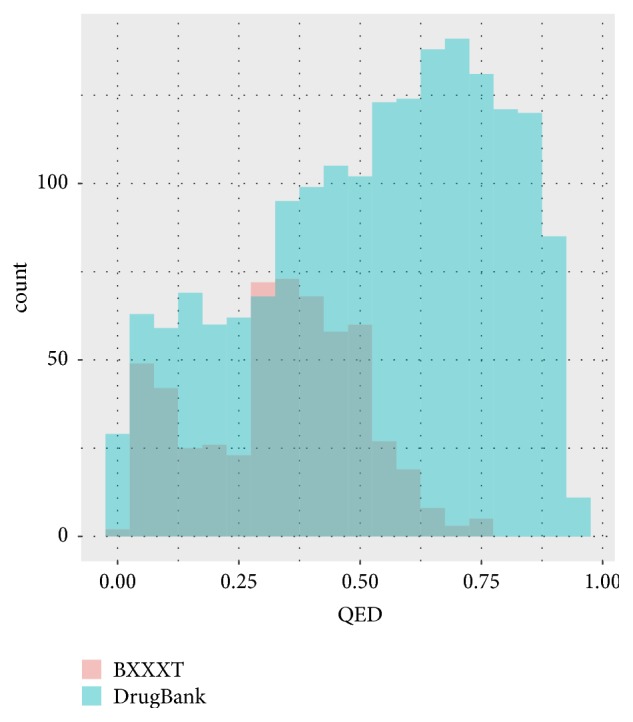
The distributions of QED values of BXXXT compounds and DrugBank drugs.

A total of 560 unique compounds were obtained from seven herbs in BXXXT. In order to characterize the structure features and assess the drug-likeness of compounds in BXXXT, a combined dataset was constructed by merging 1805 FDA-approved drugs from the DrugBank database [37] and BXXXT compounds. Then eight drug-likeness descriptors were calculated for each compound, and a principal component analysis (PCA) was employed to visually characterize the spatial distributions of the two classes of compounds.  Figure 2 shows the PCA score plot. The total variance explained by the first three principal components was 73.37%. The BXXXT and DrugBank compounds were coloured in red and blue, respectively. As seen in Figure 2, there is no clearly defined separation between the BXXXT and DrugBank compounds on the major source of variation, which means that the BXXXT compounds share the most of the chemical space with the DrugBank drugs. The distribution of the weighted composite of the other eight drug-likeness descriptors by the desirability function (QED) is shown in Figure 3. There are more than 80 percent of DrugBank drugs with the QED value greater than 0.25, and a good overlap is observed in the distributions between BXXXT compounds and DrugBank drugs. The distributions of QED of both classes are slightly skewed toward higher values. The comparison analysis of the property distributions suggests that most BXXXT compounds may be drug-like. In this work, a cutoff value of 0.25 was used to filter out the potential non-drug-like BXXXT compounds. Consequently, 402 BXXXT compounds with QED values greater than 0.25 and their corresponding targets were remained for subsequent analysis.

### 3.2. The Formula Target Profiling

After removing compounds without any interactions, a compound-target dataset of 3582 interactions between 144 compounds and 1850 targets was obtained. Then each gene was scored by (2), and a total of 140 protein targets with GS value greater than zero were selected as the formula targets (Table 1). Similarly, each compound was ranked according to its CS value calculated by (3), and the top 72 compounds that covered all of the formula targets were considered as representative compounds in BXXXT. All representative compounds and their CS values are available in Supporting Information Table S4.  Table 2 summarizes the numbers of the formula targets and representative compounds of each herb in BXXXT. We can see that the numbers of representative compounds are all below 20, while the numbers of targets vary widely from 37 to 126.

To compare the mechanisms of BXXXT and approved drugs for the treatment of according disease, a simple overlap analysis was performed on their target profiling, respectively. As a result, there were 16 common target genes shared between BXXXT and approved drugs, and the number of common targets for colitis, DM, and GC was 8, 6, and 3, respectively. As can be seen from Table 3, most of common targets were shared by colitis drugs, and three colitis drugs (mesalazine, sulfasalazine, and balsalazide) all had more than 3 common targets with BXXXT. These findings suggest that BXXXT may have the similar mechanisms of action of these drugs for the treatment of colitis.

### 3.3. Multilayer Network Analysis

In our investigation, multilayer networks were constructed based on the formula targets and disease genes, respectively. The general network properties of these networks are summarized in Table 4. We can see that the CNs were more sparse than SPENs and NENs because no extension methods were applied, while, for each level network, the formula network shows very different from the corresponding disease networks in terms of most of network parameters. According to our extension technique, the set of CN genes is a subset of that of SPEN genes, and the set of SPEN genes is a subset of that of NEN genes. SPENs were most dense by adding the close genes into CNs. The degree of node overlap between networks was measured for each level (Table 5). As can be seen in Table 5, the similarity scores are all greater than zero, which means that BXXXT targets the disease genes directly for each level. There was a good overlap between BXXXT nodes and disease nodes. As expected, higher similarity scores were observed at SPEN level and NEN level. The largest similarity score (0.8273) was observed between BXXXT and colitis at NEN level, which means that 82.73% of colitis-NEN genes were also included in BXXXT-NEN. Then the diseases were ranked according to their similarity scores as follows: colitis > GC > DM. These findings suggest that the therapeutic effects of BXXXT may be associated with the ability of the formula compounds targeting on both the disease genes directly and the genes closely connected to disease genes indirectly, and the most highly associated disease is colitis.

### 3.4. Functional Annotations

#### 3.4.1. GO Enrichment Analysis

**Figure 4:**
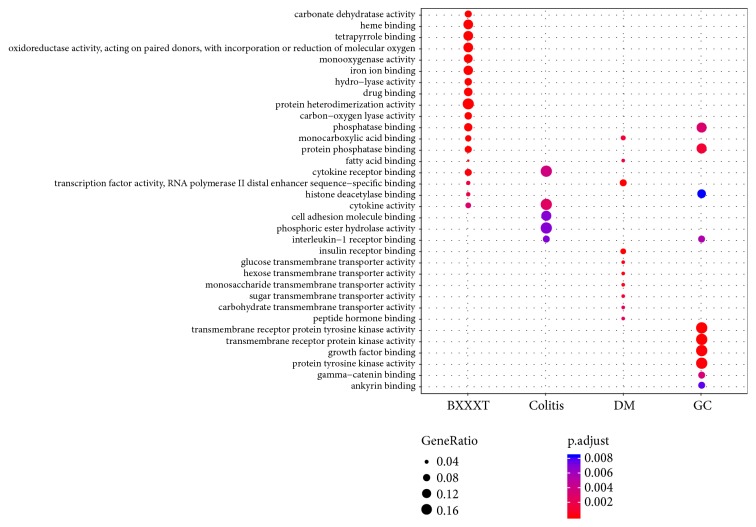
The top 10 GO terms associated with BXXXT targets and disease genes for MF ontology.

**Figure 5:**
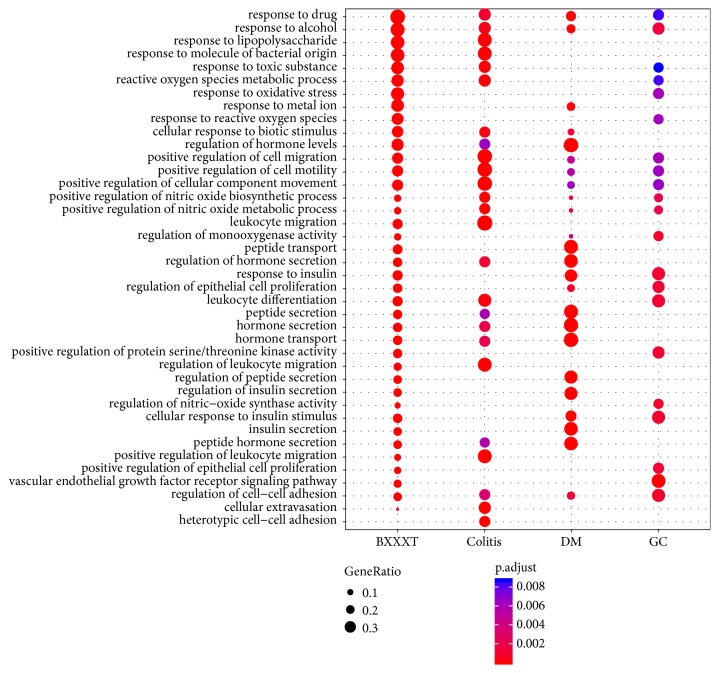
The top 10 GO terms associated with BXXXT targets and disease genes for BP ontology.

**Figure 6:**
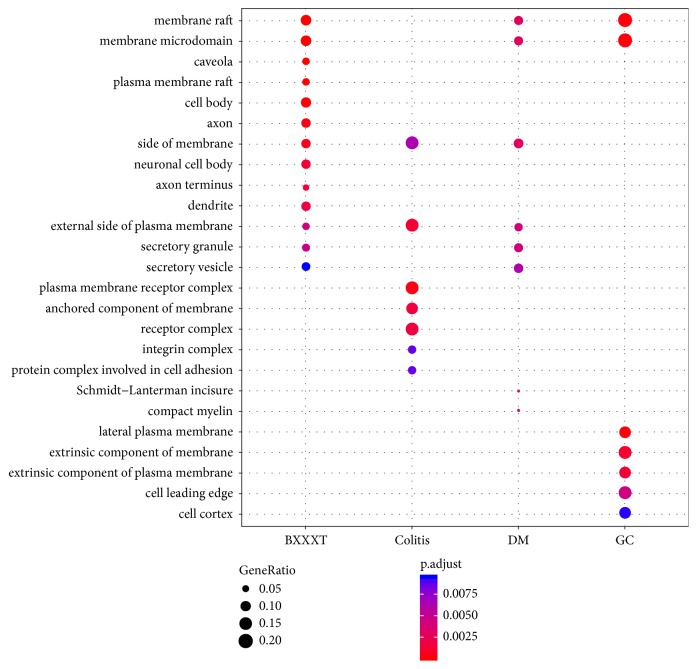
The top 10 GO terms associated with BXXXT targets and disease genes for CC ontology.

GO enrichment analysis was carried out for the identification of common features of the formula targets and disease genes in terms of GO annotation, respectively. The detailed results of enrichment analysis for three ontologies (MF, BP, and CC) are available in Supporting Information Tables S5–S7. The number of GO terms that are significantly associated with the formula targets for MF, BP, and CC is 84, 1515, and 21 (adjusted* P value* < 0.01), respectively. By comparison with the results of disease genes, we found that the formula shared many significantly associated GO terms with the diseases for each ontology. The number of common significantly associated MF GO terms between the formula and colitis, DM, and GC is 2, 3, and 3, respectively. Similarly, the numbers of common significantly associated BP GO terms and CC GO terms between the formula and colitis, DM, and GC are 292 and 2, 431 and 6, and 119 and 2, respectively. Figures 4–6 show the top 10 GO terms associated with the formula and diseases for three ontologies, respectively. For MF ontology, the top 2 GO terms (GO:0005125 and GO:0005126) associated with colitis are cytokine-related terms, and they are also significantly associated with BXXXT. Although there were a small number of significant terms for CC ontology and several common terms between BXXXT and diseases were still observed. Good overlaps among all categories were found for BP ontology, which suggested that BXXXT treats multiple diseases probably by regulating some potential shared biological processes among diseases.

**Figure 7:**
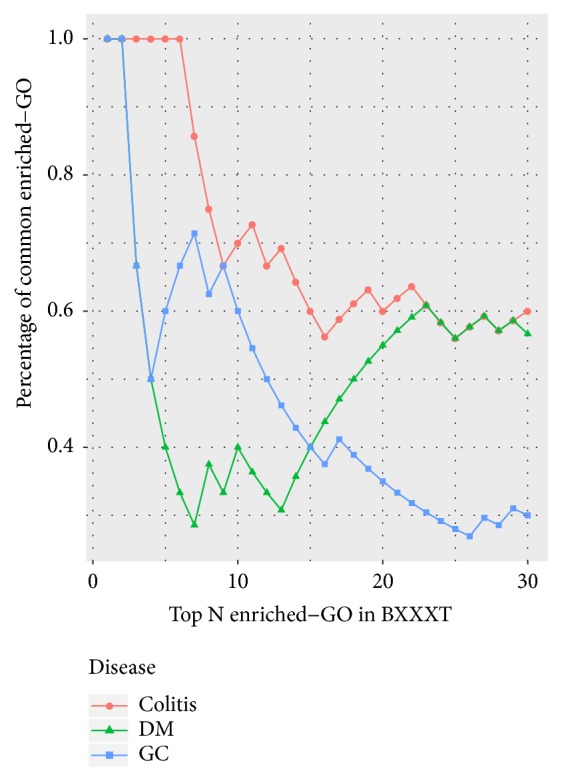
The cumulative distribution of percentages of common terms from top *N*  (30) enriched-GO terms in BXXXT for each disease.

Furthermore, the cumulative distribution of the percentages of common enriched-GO terms was used as a measure for comparing the degree of association between BXXXT and different diseases [70]. The motivation of using this measure is that a disease that has a higher probability of association with BXXXT will share more common terms in the top-k enriched-GO terms of BXXXT. The significantly enriched-GO terms were combined and sorted in ascending order according to adjusted* P values* for each group, and the percentage of common terms from top *N* (30) enriched terms in BXXXT for each disease is presented in Figure 7. We observe that the cumulative curve of colitis achieved the largest percentage value at each value of *N*. The area under the cumulative curve (AUCC) was calculated using trapezoidal integration. The AUCC value was 20.43, 14.57, and 13.61 for colitis, DM, and GC, respectively. These findings highlight the strong association between BXXXT targets and colitis genes in terms of GO.

#### 3.4.2. KEGG Pathway Enrichment Analysis

**Figure 8:**
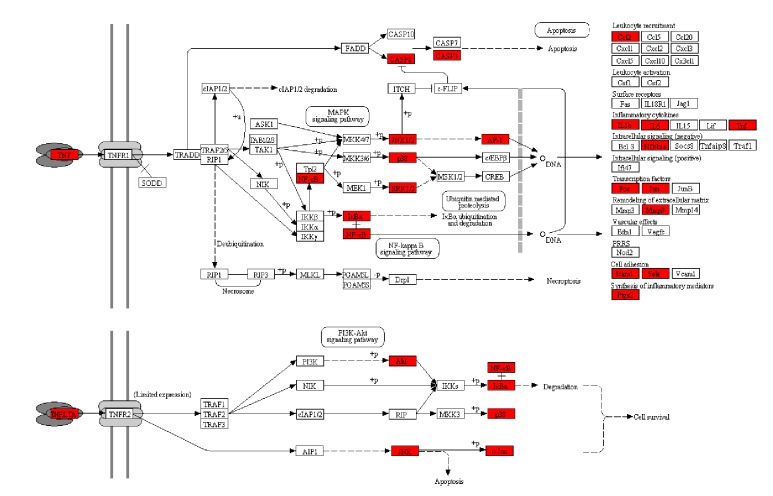
Regulations of BXXXT on TNF signaling pathway. Red boxes represent BXXXT targets.

The formula targets were mapped onto KEGG pathways, and a pathway enrichment analysis was performed to identify the biological pathways regulated by BXXXT. In this section, in order to elucidate the basic biological process, we ignored the KEGG pathway sections of human disease and drug development. As a result, 22 key pathways with adjusted* P value* < 0.01 were found to be significantly associated with BXXXT (Table 6). BXXXT acts on a large fraction of pathways in signal transduction and endocrine system. The top significantly affected pathway is tumor necrosis factor (TNF) signaling pathway (Figure 8), and the formula targets were coloured in red. As can be seen in Figure 8, a total of 20 genes are regulated by BXXXT. As a critical cytokine, TNF is associated with various physiological and pathological processes such as coagulation, cell proliferation and apoptosis, and proinflammation [71]. BXXXT has effects on two TNF receptors (TNFR1 and TNFR2). Through complex signaling cascades and networks, these effects lead to the regulations of nuclear factor-kappa B (NF-Kappa B), activation protein-1 (AP-1) via the jun nh2-terminal kinase (JNK), and caspase family members (CASP8 and CASP3). Some of our predictions have been supported by recent experimental evidence [29]. Chen's experiment [29] found that BXXXT significantly regulated the level of TNF-*α*, IL-1*β*, IL-17, IL-23, COX-2, p-p65, MPO, SOD, and Nrf2 in colorectal tissue of dextran sulfate sodium-induced chronic ulcerative colitis mice. TNF mediates many genes that are involved in other pathways including NF-kappa B signaling pathway [29], PI3K-Akt signaling pathway, and apoptosis pathway leading to cell survival and death [72]. Table 6 shows that these crossed pathways are also significant (adjusted* P value* < 0.01), implying that BXXXT exhibits multifunctional biological activities by regulating multiple pathways.

Moreover, the associations between these key pathways and disease genes were investigated in the same way, and the results are also presented in Table 6. We can find that the number of significantly overrepesented pathways for colitis, DM, and GC was 5, 9, and 10, respectively. This means that each key pathway is enriched by both the formula targets and disease genes. BXXXT can be used to treat multiple diseases probably by regulating some common biological pathways. The results of recent experiment [73] showed that the therapeutic mechanism of action of BXXXT was via insulin signaling pathway, which were concordant with our predictions (Table 6).

**Figure 9:**
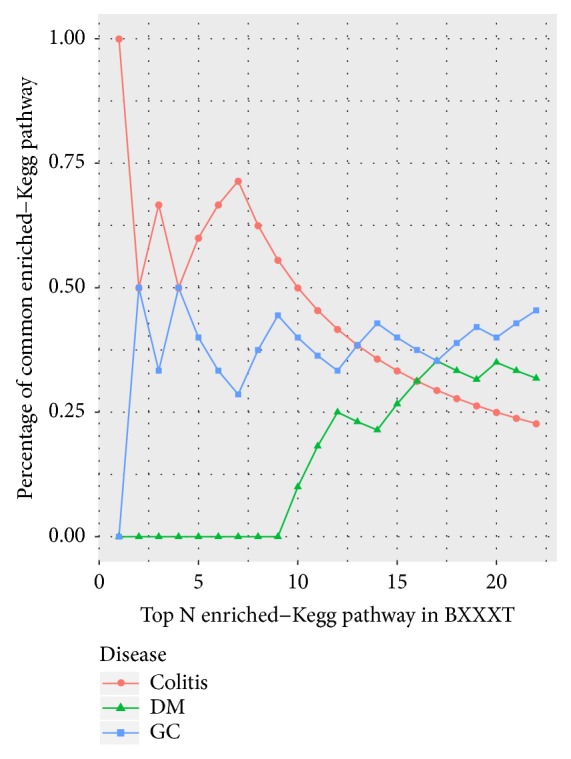
The cumulative distribution of percentages of common terms from top* N* enriched pathways in BXXXT for each disease.

Similarly, the cumulative distribution of the percentages of common enriched pathways (Figure 9) shows that the curve of colitis achieved the largest percentage value in top 12 enriched pathways, and the AUCC value was 9.52, 3.40, and 8.08 for colitis, DM, and GC, respectively. These findings further confirmed the strong association between BXXXT targets and colitis genes in terms of KEGG pathway.

### 3.5. Network-Based Proximity Analysis

To measure relevance between the formula targets and disease genes, SPD and RWR based scores were calculated. The permutation test was used to assess the significance of relevance scores. For each disease, we kept the original formula targets and randomly selected the same number of genes as that of the disease in PPI network, and a shuffled version score was calculated. This procedure was repeated independently 2000 times, and the* P value* of the original relevance score was derived thereafter.  Table 7 summarizes the results of network-based proximity analysis. We can see that the average SPD score is significantly smaller between the formula targets and disease genes than between the formula targets and randomly selected genes (*P value *< 0.01) for each disease, while the average RWR score is significantly larger. The results highlight the specificity of BXXXT for treating these diseases.

### 3.6. Network Module Analysis

**Figure 10:**
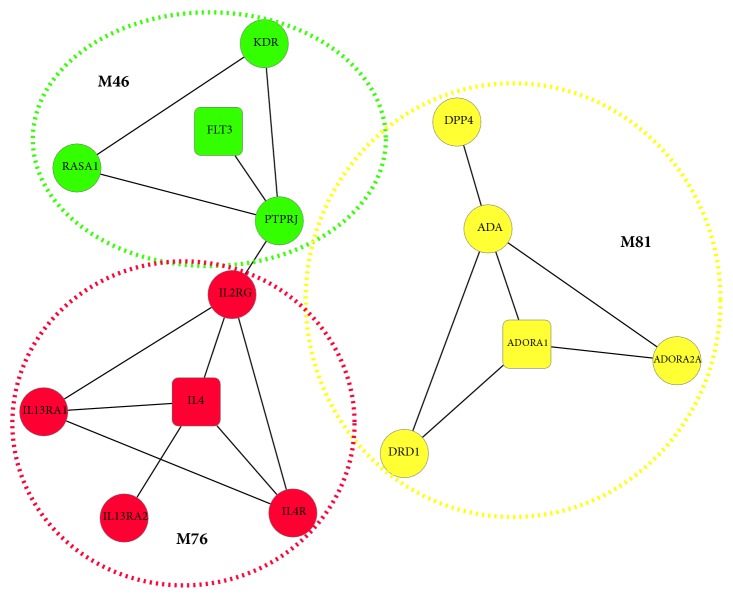
Example of representative network modules.

In this study, we compared five different network module detection methods based on BXXXT- SPEN, and Table 8 presents the results. We found that Mofinder achieved the highest* MS* score and the moderate number of modules. Then the modules resulting from Mofinder were saved and further analyzed. These modules could be considered as primarily pharmacological units of BXXXT. The association between each module and each disease genes was scored using the RWR method. The detailed results are available in Supporting Information Table S8. The top associated module where there were comembers of the formula targets for each disease is plotted in Figure 10. As shown in Figure 10, the top associated module with colitis (M76), DM (M81), and GC (M46) was coded in red, yellow, and green, respectively. The formula targets were shaped by squares and the intermediate genes were shaped by circles. We found that M76 mainly consists of the interleukin family cytokines. These cytokine genes including IL4, IL13RA2, IL13RA1, IL4R, and IL2RG have been shown to play key roles in mediating the inflammation process [74]. M81 mainly consists of the adenosine related genes, and these proteins were found to be associated with metabolic diseases by many studies [75–86]. As an intermediate gene, the well-known target (DPP4) was included in M81 by our extending approach. A relatively new class of diabetes drugs have been successfully developed by blocking DPP4 such as sitagliptin, vildagliptin, and saxagliptin [87]. In M46, protein FLT3, RASA1, and PTPRJ are considered as key signaling nodes to coordinately regulate various cellular processes including cellular proliferation, differentiation, mitotic cycle, and oncogenic transformation [88–95]. KDR acts as a major growth factor for endothelial cells, allowing regulation of endothelial proliferation, migration, and survival[96], which is associated with a variety of tumor types [97, 98].

**Figure 11:**
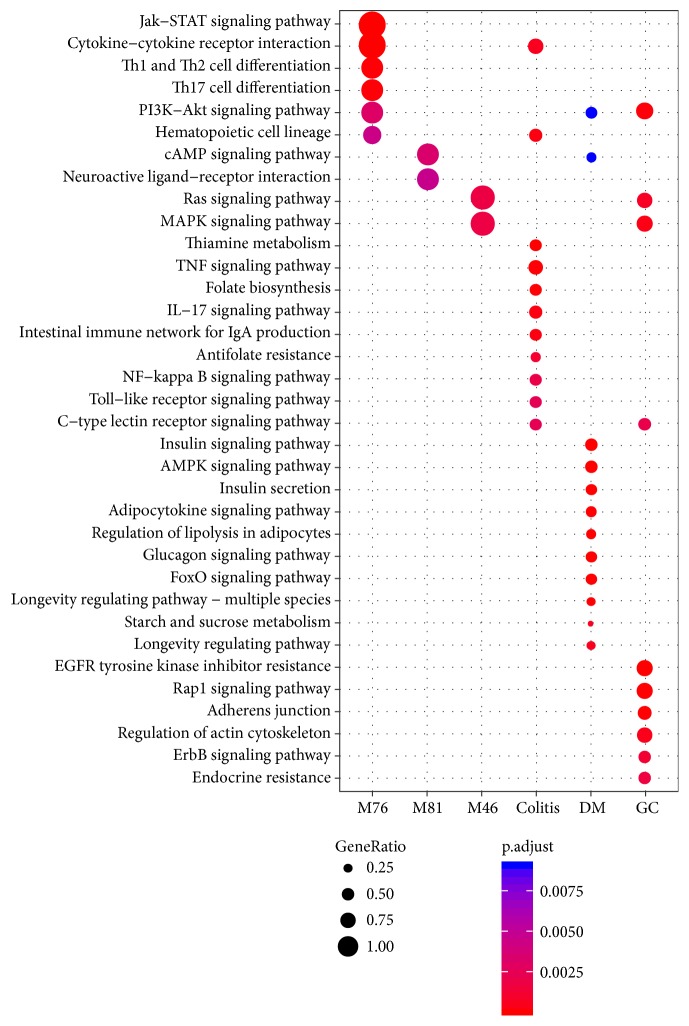
KEGG pathways significantly enriched with the module genes and disease genes.

KEGG pathway enrichment analysis was implemented to identify the pathways regulated by the above modules and disease genes, respectively.  Figure 11 shows that a total of 10 pathways were found to be significantly associated with the module genes (adjusted* P value* < 0.01). The most relevant functions and pathways for M76, M81, and M46 were related to Jak-STAT signaling pathway and cytokine-cytokine receptor interaction, cAMP signaling pathway, and Ras signaling pathway, respectively. Most of these pathways were also significantly associated with the disease genes. Such good overlaps suggest that the involved pathways partly explain the molecular mechanisms of BXXXT in treating multiple diseases.

### 3.7. Combinatorial Rules of BXXXT

**Figure 12:**
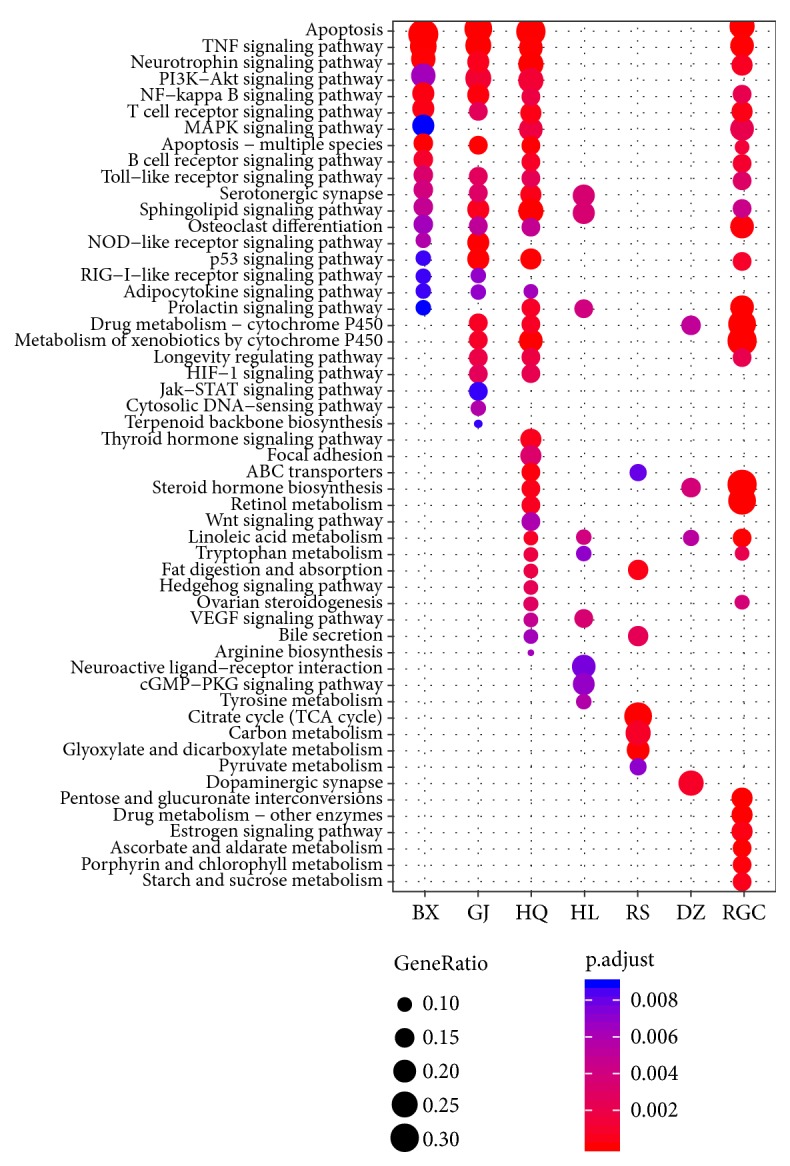
KEGG pathways significantly enriched with the targets of BXXXT herbs.

Considering that BXXXT was developed for treating digestive inflammations according to the theory of original context, colitis is regarded as a major indication for BXXXT. In this section, to better elucidate combinatorial rules of BXXXT for treating colitis, the mechanism of action of each herb in the formula was analyzed in the same way. First, targets of each herb were assessed using (2), and top 30 ranked targets according to their* GSs* were selected as the core targets of herb, named herb targets. Second, KEGG pathway enrichment analysis was performed for herb targets to identify the biological pathways of each herb. Third, to understand the role of herbs in BXXXT for treating major disease, the relevance between herb targets and colitis genes was analyzed based on RWR scores. The herb targets of BXXXT are available in Supporting Information Table S9. A simple overlap analysis between herb targets and colitis genes shows that the most common target (TNF) is shared by four herbs, which indicates the synergistic effect of the formula compositions. The significantly overrepresented pathways with adjusted* P value* < 0.01 for each herb were identified (Figure 12). The number of significantly enriched pathways for BX, GJ, HL, HQ, RS, DZ, and RGC was 18, 22, 9, 34, 7, 4, and 27, respectively. There is a large overlap of significantly enriched pathways (adjusted* P value* < 0.01) among BX, GJ, HQ, and RGC, and a total of 31 pathways are regulated by multiple herbs. These findings suggest that the synergistic strength of multiple herbs in BXXXT may be associated with common regulated pathways.

**Figure 13:**
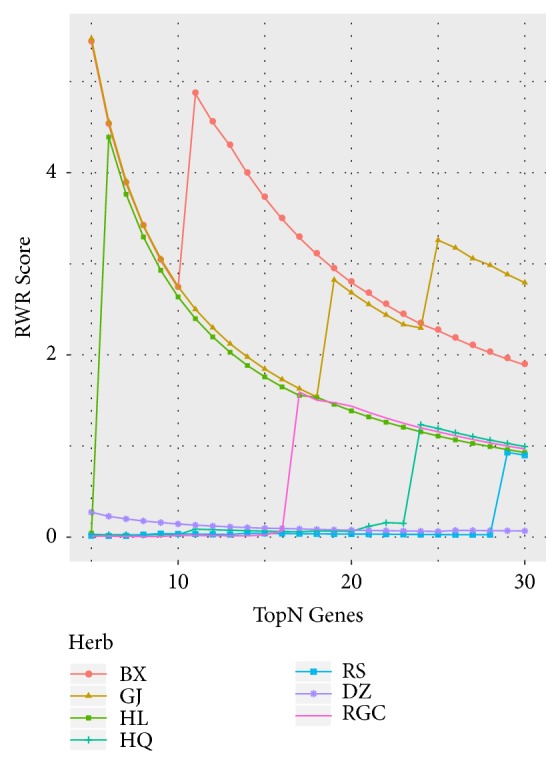
The cumulative distribution curve of RWR score of top* N* targets for each herb.

**Figure 14:**
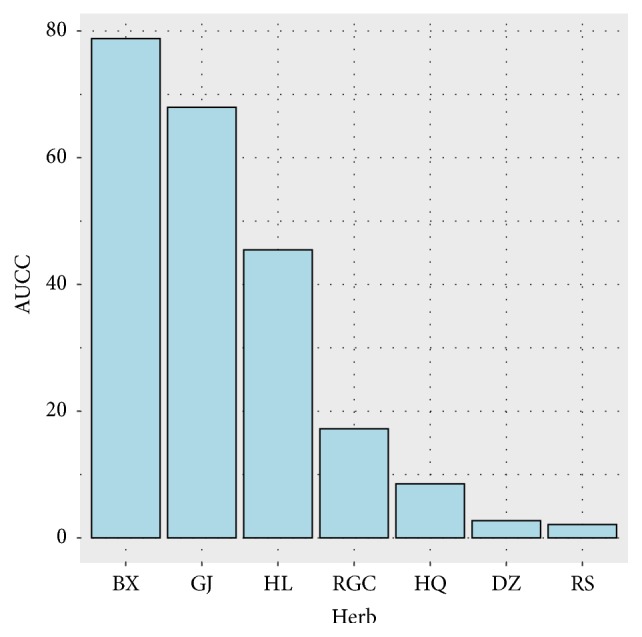
AUCC of RWR score curve for each herb.

RWR based score was calculated to measure relevance between colitis genes and herb targets for each herb. It should be noted that targets with greater* GS* values could be more important. Therefore, in this procedure, herb targets were sorted in descending order according to their* GSs*. Then each target's RWR score was sequentially calculated, and Figure 13 presents the cumulative distribution curve of RWR score of top* N* targets for each herb. We can see that BX, GJ, and HL achieved higher scores, while RS and DZ showed smaller relevance with the disease. TCM prescriptions are usually based on the principle of “Jun-Chen-Zuo-Shi”. Herbs in the formula play different roles during treatment. According to the theory of original context, “Jun” herb treats the disease directly, while others can treat the disease indirectly. In BXXXT, BX acts as “Jun” herb, GJ, HL, and HQ are “Chen” herbs, DZ and RS are “Zuo” herbs, and “RGC” serves as “Shi” herb. After calculating AUCC of RWR score curve for each herb (Figure 14), we found that AUCC value was increased in the following order: BX>GJ>HL>RGC>HQ>DZ>RS, which shows better concordance with the theory of original context except RGC. It is particularly surprising that RGC achieved higher relevance with colitis. This is probably due to the fact that this herb contains a variety of triterpene saponins that have shown a wide range of corticosteroid-like activities, and many studies have reported their anti-inflammatory actions [99–104].

## 4. Limitations and Future Work

In this study, we presented a computational approach for identification of the molecular mechanisms of BXXXT. Although it has achieved encouraging results, there are some limitations. First, our results largely rely on available data sources including herbal chemical identifications, chemical-protein interactions, and protein-protein interactions. Due to the lack of standardization and full assessment, these retrieves may contain many false positive and false negative interactions. On the other hand, the exact spectrum of compounds of TCM herbs is not defined, resulting in bias and incomplete inferences. Therefore, an updated and better validated data source may achieve more reliable and robust network models. Second, only three diseases (colitis, DM, and GC) were discussed because of their highly representative features of BXXXT treatment in clinical practices. However, TCM treatment is based on TCM syndromes that may be diverse and complicated even in the same disease or show the same characteristics in the different diseases. Although our results suggested that some common biological processes among diseases may be the potential mechanisms of BXXXT for the treatments of multiple diseases, these associations need to be further investigated in order to elucidate the biological basis of TCM syndromes. Third, common functional terms (GO terms and KEGG pathways) were identified by enrichment analysis to elucidate the relationship between the formula and disease. However, the exact associations were not investigated. It is necessary to address these association patterns in future work to achieve more accurate and more meaningful inferences. Finally, cautious interpretations should be made as our approach presented here is based on in silico analysis. Therefore, further experimental validation on the prediction of network pharmacology is needed to support the presented hypothesis in the future work.

## 5. Conclusions

The current work applied a network pharmacology approach to the case of BXXXT formula, in order to elucidate its therapeutic mechanisms in treating multiple diseases. To maintain a reasonable level of reliability, a network-level investigation that integrated drug-likeness screening, target prioritizing, and multilayer network extending was conducted. We focused on 140 core formula targets, and three representative BXXXT diseases (colitis, DM, and GC) were discussed. Our main findings are as follows.

(1) After constructing multilayer networks, a good overlap between BXXXT nodes and disease nodes was observed at each level. The degree of similarity between BXXXT and colitis achieved the highest score. Moreover, the network-based proximity analysis shows that the relevance between the formula targets and disease genes was significant according to SPD and RWR based scores for each disease. These results suggest that the formula targets are significantly close to disease genes in the PPI network, and the therapeutic effects of BXXXT may be addressed by targeting on both the disease genes directly and the genes closely connected to disease genes indirectly.

(2) The pathway enrichment analysis shows that there were 22 key pathways significantly associated with BXXXT, and the top significantly affected pathway was TNF signaling pathway. The analysis of the cumulative distribution of percentages of common enriched terms (GO terms and KEGG pathways) further confirmed the good association between BXXXT targets and disease genes.

(3) Our network module analysis has taken into account density and functional homogeneity simultaneously. The therapeutic effects of BXXXT were likely addressed by regulating a combination of targets in a modular pattern.

(4) The synergistic effects among BXXXT herbs were highlighted by elucidating that multiple herbs act on the same targets and the same pathways. Besides, the traditional roles of individual herbs in BXXXT formula were effectively interpreted based on the molecular level. It provides a new way to shed lights on the theory of “Jun-Chen-Zuo-Shi” of TCM from a network perspective.

In summary, the proposed approach is an effective strategy to understand the mechanisms of action and combinatorial rules of BXXXT formula. Also, the results of this work may facilitate generating hypothesis to drug development based on BXXXT and enable further research in a more time-saving and cost-effective manner.

## Figures and Tables

**Table 1:** The core targets of BXXXT.

140 Genes (gene symbol)
ABCA1, ACHE, ADORA1, PARP1, ADRA2C, AHR, AKT1, ALB, AKR1B1, ALOX5, ALOX15, BIRC5, BAX, BCHE, CCND1, BCL2, BDNF, CA1, CA2, CA3, CA4, CA5A, CA6, CA7, CA9, CA12, CASP3, CASP8, CASP9, CAT, CDK1, CDK2, COMT, MAPK14, CSN1S1, CYP1A1, CYP1A2, CYP1B1, CYP2B6, CYP2C19, CYP2C9, CYP2D6, CYP2E1, CYP3A4, CYP19A1, DECR1, NQO1, DRD2, DRD3, DRD4, EGFR, ESR1, ESR2, F3, FASN, FLT3, FOS, GCG, GSK3B, GSTP1, HIF1A, HMGCR, HMOX1, HRH1, HRH2, HTR1A, HTR7, ICAM1, IFNG, IL1B, IL4, IL6, CXCL8, INS, INSR, EIF6, JUN, KCNH1, LCK, LPL, MAOA, MAPT, MMP1, MMP2, MMP9, MPO, ABCC1, NEU2, NFE2L2, NFKB1, NFKBIA, NOS1, NOS2, NOS3, ABCB1, PLA2G1B, PLAU, POLB, PON1, PPARA, PPARD, PPARG, PREP, PRKCA, MAPK1, MAPK3, MAPK8, PTGER3, PTGS1, PTGS2, PTPN1, RB1, RELA, CCL2, SELE, SOD2, SRC, SREBF2, SULT1A1, TH, TNF, TOP2A, TP53, TYR, UROD, VEGFA, XDH, AKR1C3, MGAM, SLCO1B1, CA5B, CA14, SLCO1B3, UGT1A10, UGT1A8, UGT1A7, UGT1A9, UGT1A4, UGT1A1, ABHD6

**Table 2:** The distribution of core targets in BXXXT herbs.

Herb	Number of representative compounds	Number of core targets
BX	10	44
GJ	13	37
HL	7	105
HQ	10	94
RS	7	63
DZ	15	107
RGC	18	126

**Table 3:** Common targets shared between BXXXT and approved drugs.

Common targets	Database ID	Drug	Disease
TNF	DB00065	Infliximab	colitis
PTGS2, PTGS1, ALOX5, PPARG, MPO	DB00244	Mesalazine	
ALOX5, PTGS2, PTGS1, PPARG, PLA2G1B	DB00795	Sulfasalazine	
PPARG, PTGS2, PTGS1, ALOX5	DB01014	Balsalazide	
IFNG	DB01250	Olsalazine	
TNF	DB06674	Golimumab	
INSR,RB1	DB00030	Insulin Human	DM
INSR	DB00046	Insulin Lispro	
INSR	DB00047	Insulin Glargine	
INSR,RB1	DB00071	Insulin Pork	
MGAM	DB00284	Acarbose	
PPARG	DB00412	Rosiglitazone	
MGAM	DB00491	Miglitol	
PPARG	DB00731	Nateglinide	
PPARG	DB00912	Repaglinide	
ABCA1	DB01016	Glyburide	
PPARG	DB01067	Glipizide	
VEGFA	DB01120	Gliclazide	
PPARG	DB01132	Pioglitazone	
PPARG	DB01252	Mitiglinide	
INSR	DB01306	Insulin Aspart	
INSR	DB01307	Insulin Detemir	
INSR	DB01309	Insulin Glulisine	
MGAM	DB04878	Voglibose	
TOP2A	DB00997	Doxorubicin	GC
EGFR	DB05448	PX-12	
BCL2	DB05457	OSI-7904L	

**Table 4:** The general network properties of multilayer networks.

Level	Network parameter	BXXXT	Colitis	DM	GC
CN	Number of nodes	135	27	95	27
	Number of isolated nodes	49	22	55	13
	Average degree	4.13±5.75	0.3±0.67	0.86±1.52	1.63±2.1
	Average betweenness	32.9±71.86	0±0	8.16±26.43	2.48±5.92
	Density	0.0308	0.0114	0.0092	0.0627
	Cluster coefficient	0.2984	1.0000	0.2039	0.4225
	Diameter	6	1	7	4
	Shortest path	2.6333	1.0000	3.2270	2.0000
SPEN	Number of nodes	1083	174	722	160
	Number of isolated nodes	0	0	0	0
	Average degree	35.89±37.83	14.98±11.49	32.04±30.54	21.34±14.12
	Average betweenness	779.85±1964.53	117.51±173.57	523.02±1010.8	83.38±120.72
	Density	0.0332	0.0866	0.0444	0.1342
	Cluster coefficient	0.1642	0.2472	0.1975	0.2942
	Diameter	5	5	6	4
	Shortest path	2.4415	2.3584	2.4508	2.0487
NEN	Number of nodes	5374	1187	2439	2575
	Number of isolated nodes	0	0	0	0
	Average degree	46.56±73.94	40.49±51.12	43.67±57.71	44.77±63.8
	Average betweenness	4334.96±25925.21	830.27±3262.88	1923.32±6887.23	1813.74±13825.69
	Density	0.0087	0.0341	0.0179	0.0174
	Cluster coefficient	0.1006	0.1974	0.1494	0.1247
	Diameter	6	7	7	6
	Shortest path	2.6136	2.4001	2.5778	2.4093

**Table 5:** Results of node similarity analysis between BXXXT-network and disease-network at each level.

Network level		Disease	
	Colitis	DM	GC
BXXXT-CN	0.2963	0.0842	0.1481
BXXXT-SPEN	0.7126	0.4806	0.6563
BXXXT-NEN	0.8273	0.7618	0.781

**Table 6:** KEGG pathways significantly enriched with targets of BXXXT.

Class	KEGGID	Description	Adjusted P value	Associated Disease (Adjusted P value)
Aging	hsa04211	Longevity regulating pathway	2.95×10^−5^	DM (5.65×10^−4^)
Carbohydrate metabolism	hsa00500	Starch and sucrose metabolism	1.99×10^−4^	DM (5.43×10^−4^)
Cell growth and death	hsa04210	Apoptosis	9.41×10^−10^	GC (4.72×10^−3^)
Cellular community	hsa04510	Focal adhesion	9.11×10^−4^	GC (2.33×10^−3^)
	hsa04520	Adherens junction	5.29×10^−3^	GC (4.62×10^−5^)
Development	hsa04380	Osteoclast differentiation	1.40×10^−7^	Colitis (5.09×10^−3^)
	hsa04917	Prolactin signaling pathway	5.84×10^−11^	DM;GC (7.92×10^−3^;6.68×10^−3^)
	hsa04915	Estrogen signaling pathway	1.43×10^−6^	GC (4.72×10^−3^)
Endocrine system	hsa04923	Regulation of lipolysis in adipocytes	1.99×10^−4^	DM (2.90×10^−6^)
	hsa04920	Adipocytokine signaling pathway	7.58×10^−4^	DM (1.52×10^−6^)
	hsa04910	Insulin signaling pathway	2.55×10^−3^	DM (1.32×10^−7^)
Immune system	hsa04621	NOD-like receptor signaling pathway	3.31×10^−10^	Colitis (6.21×10^−3^)
	hsa04620	Toll-like receptor signaling pathway	8.69×10^−9^	Colitis (2.42×10^−3^)
	hsa04668	TNF signaling pathway	3.25×10^−13^	Colitis (7.96×10^−5^)
	hsa04064	NF-kappa B signaling pathway	8.09×10^−7^	Colitis (1.95×10^−3^)
	hsa04370	VEGF signaling pathway	8.98×10^−7^	GC (4.72×10^−3^)
	hsa04068	FoxO signaling pathway	5.11×10^−6^	DM (2.00×10^−5^)
Signal transduction	hsa04150	mTOR signaling pathway	4.33×10^−5^	DM (2.11×10^−3^)
	hsa04151	PI3K-Akt signaling pathway	5.29×10^−5^	DM;GC (9.05×10^−3^;1.22×10^−4^)
	hsa04012	ErbB signaling pathway	9.33×10^−5^	GC (1.19×10^−3^)
	hsa04014	Ras signaling pathway	7.58×10^−4^	GC (7.38×10^−4^)
	hsa04015	Rap1 signaling pathway	4.20×10^−3^	GC (4.62×10^−5^)

**Table 7:** Results of network-based proximity analysis.

Method	Disease	Type	Average score	P value
SPD	Colitis	original	2.87	5.30×10^−4^
		shuffled	3.10	
	DM	original	2.92	1.13×10^−7^
		shuffled	3.14	
	GC	original	2.56	5.41×10^−6^
		shuffled	2.77	
RWR	Colitis	original	1.67	3.68×10^−12^
		shuffled	0.06	
	DM	original	0.55	1.23×10^−6^
		shuffled	0.06	
	GC	original	0.86	7.24×10^−13^
		shuffled	0.06	

**Table 8:** Comparisons of different network module detection methods.

Method	Modules	Module Size	Q	BHI	MS
FG	8	2~509	0.1992	0.1766	0.3758
MCODE	21	3~110	0.0242	0.4465	0.4707
NeMo	290	4~89	0.0523	0.3643	0.4166
MOfinder	257	4~22	0.3263	0.4611	0.7874
IPCA	819	5~45	0.3403	0.421	0.7613

## Data Availability

Supporting data and materials are available: BXXXT compounds and their targets (Table S1), disease associated genes (Table S2), and DrugBank drugs and their targets (Table S3), all representative compounds in BXXXT and their CS values (Table S4), enrichment analysis for MF ontology (Table S5), enrichment analysis for BP ontology (Table S6), enrichment analysis for CC ontology (Table S7), results of network module analysis (Table S8), and the targets of BXXXT herbs (Table S9).

## Supplementary Materials

Supplementary Materials BXXXT compounds and their targets (Table S1). Disease associated genes (Table S2). DrugBank drugs and their targets (Table S3). All representative compounds in BXXXT and their CS values (Table S4). Enrichment analysis for MF ontology (Table S5). Enrichment analysis for BP ontology (Table S6). Enrichment analysis for CC ontology (Table S7). Results of network module analysis (Table S8). The targets of BXXXT herbs (Table S9).
